# Feiyanning formula modulates the molecular mechanism of osimertinib resistance in lung cancer by regulating the Wnt/β-catenin pathway

**DOI:** 10.3389/fphar.2022.1019451

**Published:** 2022-11-29

**Authors:** Shuliu Sang, Chenbing Sun, Rongzhen Ding, Jingjie Jiang, Yang Han, Shanshan Gan, Ling Bi, Yabin Gong

**Affiliations:** ^1^ Department of Oncology, Yueyang Hospital of Integrated Traditional Chinese and Western Medicine, Shanghai University of Traditional Chinese Medicine, Shanghai, China; ^2^ Institutional Key Laboratory of Vascular Biology and Translational Medicine in Hunan Province, Hunan University of Chinese Medicine, Changsha, China

**Keywords:** Feiyanning formula, osimertinib, drug resistance, lung cancer, Wnt/β-catenin pathway

## Abstract

Feiyanning Formula (FYN), a Chinese herbal formula derived from summarized clinical experience, is proven to have anti-tumor effects in lung cancer patients. Osimertinib, a third-generation epidermal growth factor receptor-tyrosine kinase inhibitor (EGFR-TKI), can improve progression-free survival and overall survival of patients but drug resistance is inevitable. The current study evaluated the effects of FYN in osimertinib-resistant HCC827OR and PC9OR cells. FYN preferentially inhibited the proliferation and migration of HCC827OR and PC9OR cells. Moreover, FYN and osimertinib exhibited synergistic inhibitory effects on proliferation and migration. Real-time qPCR (RT-qPCR) and western blotting results indicated that FYN downregulated gene and protein levels of GSK3β and SRFS1, which are enriched in the Wnt/β-catenin pathway. Besides, FYN inhibited tumor growth and exhibited synergistic effects with osimertinib *in vivo*. Collectively, the results suggested that FYN exerted an anti-osimertinib resistance effect *via* the Wnt/β-catenin pathway.

## Introduction

Lung cancer is the most prevalent tumor globally and ranks first in cancer mortality (Siegel et al., 2022). The most common treatment for early stage lung cancer is surgery, whereas chemotherapy, radiotherapy, targeted therapy, and immunotherapy are commonly used in progressive cases (Lemjabbar-Alaoui et al., 2015). Unfortunately, most patients experience recurrence and metastasis after surgery, which foreshadows a poor prognosis (Schegoleva et al., 2021). To improve outcomes, the National Comprehensive Cancer Network recommended that histological subtypes and biomarkers be detected before treating lung cancer patients with recurrence and metastasis (Ettinger et al., 2022). As a third-generation epidermal growth factor receptor-tyrosine kinase inhibitor (EGFR-TKI), osimertinib is recommended as the first-line treatment in patients with epidermal growth factor receptor (EGFR) mutation-positive non-small cell lung cancer (NSCLC) (Maione et al., 2015). Osimertinib promotes longer progression-free survival (18.9 months vs 10.2 months) and better overall survival (38.6 vs. 31.8 months) than former generation EGFR-TKIs (Passaro et al., 2021), but the development of resistance is inevitable. In past decades, the mechanisms involved in osimertinib resistance have not been entirely expounded there has been no practical way to improve the situation. However, herbal compounds have exhibited outstanding potential therapeutic value recently.

Several recent studies have investigated the effects of Chinese herbal compounds on EGFR-TKI resistance (Hu et al., 2020; Tan et al., 2020). Feiyanning Formula (FYN) is an anti-cancer formula consisting of *Astragalus membranaceus, Ganoderma lucidum, Paris polyphylla,* and other Chinese herbs. In our previous clinical study, FYN combined with chemotherapy prolonged the survival of advanced NSCLC patients (Gong et al., 2018). Recent studies have shown that FYN can induce apoptosis in lung adenocarcinoma cells by activating the mitochondrial pathway (Zhu et al., 2021). However, the mechanism by which FYN delays drug resistance has not been reported yet.

In this study, the effects of FYN combined with osimertinib on PC9OR and HCC827OR cell proliferation and migration were investigated. FYN combined with osimertinib had synergistic inhibitory effects against the development of osimertinib resistance *in vivo*. These findings revealed the mechanisms involved in the application of FYN the treatment of NSCLC, and provide an experimental basis for clinical application.

## Materials and methods

### Herbs and chemicals preparation

FYN consists of 11 Chinese herbs (Table 1). All FYN herbs were provided by the pharmacy of Shanghai Chest Hospital, Shanghai Jiao Tong University (Shanghai, China). FYN was dried into a lyophilized powder and stored at -20 °C. In accordance with the experimental requirements, the lyophilized FYN powder was dissolved in RPMI-1640 medium (Hyclone, United States, SH30809.01), diluted to various concentrations, then passed through a 0.22-μm filter. Osimertinib (Selleck Company, United States, S7297) was dissolved in DMSO (Absin Bioscience Inc., China, abs9187), diluted to 200 mM, then aliquoted and stored at −20°C in the dark.

**TABLE 1:** FYN components.

Species	Active chemicals	Herbal name	Number of components (g)
Dried root of *Astragalus membranaceus (Fisch.)*	Isoflavanone,	Huang Qi	30
	3,9-di-O-methylnissolin,		
	Isorhamnetin,		
	Jaranol,		
	Kaempferol,		
	Quercetin,		
	7-O-methylisomucronulatol,		
	Hederagenin,		
	Formononetin,		
	Calycosin,		
	Mairin, (3S,8S,9S,10R,13R,14S,17R)-10,13-dimethyl-17-[(2R,5S)-5-propan-2-yloctan-2-yl]-2,3,4,7,8,9,11,12,14,15,16,17-dodecahydro-1H-cyclopenta [a]phenanthren-3-ol,		
	Bifendate		
Dried rhizome of *Polygonatum kingianum Coll. et Hemsl.*	4′,5-Dihydroxyflavone,	Huang Jing	30
	Apigenin,		
	Baicalein,		
	3′-Methoxydaidzein,		
	β-sitosterol,		
	Diosgenin,		
	Oroxin A,		
	Daucosterol,		
	Dioscin,		
	Methylprotodioscin, (+)-Syringaresinol-O-β-D-glucoside,		
	Liriodendrin_qt		
Dried fruit of *Cornus officinalis Sieb. Et Zucc*.	Hydroxygenkwanin,	Shan Zhu Yu	15
	Tetrahydroalstonine,		
	Mandenol,		
	Cornudentanone,		
	Ethyl linolenate,		
	Ethyl oleate (NF),		
	β-sitosterol,		
	Poriferast-5-en-3beta-ol,		
	Stigmasterol,		
	Diop,		
	Leucanthoside,		
	2,6,10,14,18-pentamethylicosa-2,6,10,14,18-Pentaene,		
	Gemin D		
Dried rhizome of *Paris polyphylla Smith var. yunnanensis (Franch.) Hand.-Mazz.*	Isorhamnetin,	Chong Lou	15
	Kaempferol,		
	Luteolin,		
	Quercetin,		
	Remerin,		
	Stigmasterol,		
	Daucosterol,		
	Dioscin,		
	Polyphyllin VII,		
	Polyphyllin I,		
	Polyphyllin II		
Dried rhizome of *Atractylodes macrocephala Koidz.*	Apigenin,	Bai Zhu	9
	Luteolin,		
	Ethyl caffeate,		
	α-Amyrin,		
	AtractylenolideII, (3S,8S,9S,10R,13R,14S,17R)-10,13-dimethyl-17-[(2R,5S)-5-propan-2-yloctan-2-yl]-2,3,4,7,8,9,11,12,14,15,16,17-dodecahydro-1H-cyclopenta [a]phenanthren-3-ol,		
	Atractylenolide Ⅲ,		
	Atractylenolide I		
Dried nest of *Polistes olivaceous (DeGeer)*	Kaempferol,	Feng Fang	9
	Ferulic acid,		
	β-sitosterol,		
	3,4,5-trihydroxy benzoic acid,		
	Supraene,		
	α-Carotene		
Dried plant of *Salvia chinensis Benth*.	Quercetin,	Shi Jian Chuan	30
	Resveratrol,		
	Oleanolic acid,		
	Ursolic acid,		
	β-sitosterol,		
	Salvianolic acid C,		
	Vanillin		
Dried skin of *Bufo gargarizans Cantor*	Cinobufagin,	Gan Chan Pi	6
	Cinobufotalin,		
	β-sitosterol,		
	Arenobufagin,		
	Bufalin,		
	Resibufogenin,		
	Deacetylcinobufotalin,		
	Bufotaline		
Dried fruit body of *Ganoderma lucidum (Leyss.exFr.) Karst*	Cerevisterol,	Ling Zhi	15
	Ganosporelactone B,		
	Methyl Ganoderic acid DM,		
	Epoxyganoderiol B,		
	Ganoderiol F,		
	Ganodermanondiol,		
	Methyl (4R)-4-[(5R,7S,10S,13R,14R,15S,17R)-7,15-dihydroxy-4,4,10,13,14-pentamethyl-3,11-dioxo-2,5,6,7,12,15,16,17-octahydro-1H-cyclopenta [a]phenanthren-17-yl]pentanoate,		
	Lucidumol A,		
	Ganoderal B,		
	Lucialdehyde B,		
	Methyl (4R)-4-[(5R,10S,13R,14R,17R)-4,4,10,13,14-pentamethyl-3,7,11,15-tetraoxo-2,5,6,12,16,17-hexahydro-1H-cyclopenta [a]phenanthren-17-yl]pentanoate,		
	(+)-Methyl ganolucidate A,		
	Ganoderic acid V,		
	Ganoderic acid X,		
	Ganoderic aldehyde A,		
	Lucialdehyde C,		
	Ganolucidic acid E,		
	Ganodermic acid T-Q,		
	Ganoderic acid Mi,		
	Ganoderic acid TR		
Dried pseudobulb of *Cremastra appendiculata (D.Don) Makino*	Quercetin,	Shan Ci Gu	15
	2-methoxy-9,10-dihydrophenanthrene-4,5-diol,		
	β-sitosterol,		
	Stigmasterol,		
	3,4,5-trihydroxy benzoic acid,		
	Daucosterol,		
	Colchicine		
Dried leaf of *Epimedium brevicomu Maxim.*	Chryseriol,	Yin Yang Huo	15
	Kaempferol,		
	Luteolin,		
	Quercetin,		
	8-Isopentenyl-kaempferol,		
	Linoleyl acetate,		
	Magnograndiolide,		
	Poriferast-5-en-3beta-ol,		
	Yinyanghuo A,		
	Icariin,		
	25-epicampesterol,		
	Anhydroicaritin,		
	Yinyanghuo C,		
	Yinyanghuo E		

### Cells and cell culture

HCC827 and PC9 cells originally obtained from the Cell Bank of Chinese Academy of Sciences (Shanghai, China) were cultured in RPMI 1640 medium supplemented with 10% fetal bovine serum (Gibco, United States, 10091148) and 1% penicillin–streptomycin (HyClone, United States, SV30010) in a humidified incubator (Thermo Fisher, United States) with 5% CO_2_ at 37°C. All cell types were verified by short tandem repeat profiling and were examined every 6 months for *Mycoplasma*.

### Establishment and identification of osimertinib-resistant cell line lines

PC9 and HCC827 cells were cultured in T25 flasks at logarithmic growth stages, then osimertinib was added to establish osimertinib-resistant cell lines. PC9OR and HCC827OR cells were established in our laboratory by exposing them to stepwise and incremental concentrations of osimertinib in the range of 5–3000 nM (Table 2).

**TABLE 2:** Time of cultivation with each dose.

Concentration (nM)	Time (days)
5	7
10	7
20	15
30	15
100	15
500	15
1000	20
2000	20
3000	20

### Cell viability assay

Cell proliferation was assessed *via* the CCK-8 assay. PC9OR, HCC827OR, PC9, and HCC827 cells in logarithmic growth phase were seeded into 96-well plates at a density of 5 × 10^3^ per well, and treatments (FYN, osimertinib and FYN combined with osimertinib) were applied the next day. Five replicate wells were prepared for each group. After treatment, OD values at 450 nm were measured in each well after the addition of 10 μl CCK-8 reagent for 1–4 h without refreshing the media. The inhibition ratio was calculated *via* the following formula:

$$Inhibition\;ratio\;\left(\%\right)=\left(1-{OD}_{treatment}\;/{OD}_{control}\right)\times 100\%$$

The IC_50_ values of osimertinib were calculated using GraphPad Prism 8.0 software.

### Synergistic effect analysis

Combination treatments can result in synergistic, additive, antagonistic, or potentiative effects. These effects were evaluated by calculation of the combination index (CI) in accordance with the Chou-Talalay method. Data were analyzed using CompuSyn software (CompuSyn Inc.), and CI 0.85 to 0.90 = slight synergism, CI 0.70 to 0.85 = moderate synergism, CI 0.30 to 0.70 = synergism, CI 0.10 to 0.30 = strong synergism, and CI < 0.10 = very strong synergism.

### Colony formation assay

PC9OR and HCC827OR cells were seeded into 6-well plates at a density of 1000 cells per well for 24 h before treatment. After 14 days, all wells were fixed with 4% paraformaldehyde (Biosharp Co., Ltd., China, BL539A) and stained with crystal violet (Beyotime Inc., China, C0121). After drying, the stained cells were imaged, and stained colonies containing >10 cells were counted.

### Wound healing assay

PC9OR and HCC827OR cells were inoculated in 6-well plates at 5×10^5^ per well. When cells reached 90% confluence a horizontal scratch was made with a 200-μl pipette tip. After washing away floating cells with PBS, cells were treated for 24 h. Images were obtained using an inverted microscope (Leica DMI3000B, Leica Microsystems, Inc.) at different timepoints to measure scratch width.

### Transwell assay

PC9OR and HCC827OR cells were diluted to 2 × 10^4^ per well in serum-free 1640 medium. The cells were then seeded into the upper chambers of transwell plates and different treatments were applied for 24 h. Medium containing 15% fetal bovine serum was added to the lower chamber to enhance cell migration. After 24 h, the cells were fixed with 4% paraformaldehyde and stained with crystal violet. An inverted microscope was used for image acquisition.

### Real-time qPCR (RT-qPCR) array

The Trizol reagent (Invitrogen, Thermo Fisher Scientific Inc., United States, 15596018) was used to extract total RNA from cells. The RNA was then reverse transcribed into cDNA using a cDNA synthesis kit (Bio-Rad, United States, 1708891), and gene expression profiles were analyzed by RT-qPCR array in accordance with the manufacturer’s instructions. mRNA expression levels were calculated using the 2^−ΔΔCq^ method, and GAPDH was used as an internal reference. All primers were purchased from Sangon Biotech, and were summarized in Supplementary Table S1.

### Western blotting

HCC827OR and PC9OR cells were treated with FYN at IC_50_ for 48 h to determine protein expression levels. The cells were then lysed in ice-cold RIPA buffer (Beyotime Inc., China, P0013B), and a BCA Protein Assay Kit (Beyotime Inc., China, P0010) was used to quantify protein concentrations. Total proteins in different groups were subjected to SDS-PAGE gel separation, then transferred to polyvinylidene difluoride membranes (Merck Millipore). After blocking with 5% skim milk powder at room temperature for 2 h, the membranes were incubated with primary antibodies at 4 °C overnight. Secondary antibodies were then added, and the preparations were incubated for 2 h. The probed membranes were developed with ECL solution (Sangon, Shanghai, China, C510043-0100). Primary antibodies against SRSF1 (Cat# 14908), GSK3B (Cat# 5558), β-catenin (Cat# 8480), and GAPDH (Cat# 5174) were acquired from Cell Signaling Technology. The primary antibody against PCNA (Cat# sc-56) was acquired from Santa Cruz Biotechnology.

### Tumor xenograft

Female nude mice aged 4 weeks were purchased from the Shanghai SLAC Animal Laboratory (China). After acclimatization for 1 week, 1 × 10^7^ HCC827OR cells were resuspended in 200 µl of PBS then injected subcutaneously under the left armpit of each mouse. When the mean tumor volume of all mice was approximately 100 mm^3^, the mice were divided into control group, FYN group, osimertinib group, and FYN with osimertinib combination group. The preparations applied to all four groups were administered *via* oral gavage once each day. The control group was administered water. The FYN group was administered 131.86 mg FYN powder per mouse. The osimertinib group was administered 0.1 mg osimertinib per mouse. The combination group was administered 131.86 mg FYN powder and 0.1 mg osimertinib per mouse. Body weights and tumor volumes were recorded daily. Tumor volumes were measured with a digital caliper and calculated as 0.5 × length × width^2^, and the results were presented in Supplementary Table S2. At the endpoint of the experiment, the tumors were weighted and the organs were fixed with 4% paraformaldehyde, embedded in paraffin, and stained with hematoxylin and eosin.

### Statistical analysis

Data were analyzed by GraphPad Prism 8.0, and represented as means ± standard deviation. Differences between groups were assessed *via* Student’s *t*-test or analysis of variance. Differences were considered statistically significant when *p* < 0.05.

## Results

### FYN preferentially inhibited cell proliferation and migration in osimertinib-resistant lung cancer cell lines

**FIGURE 1:**
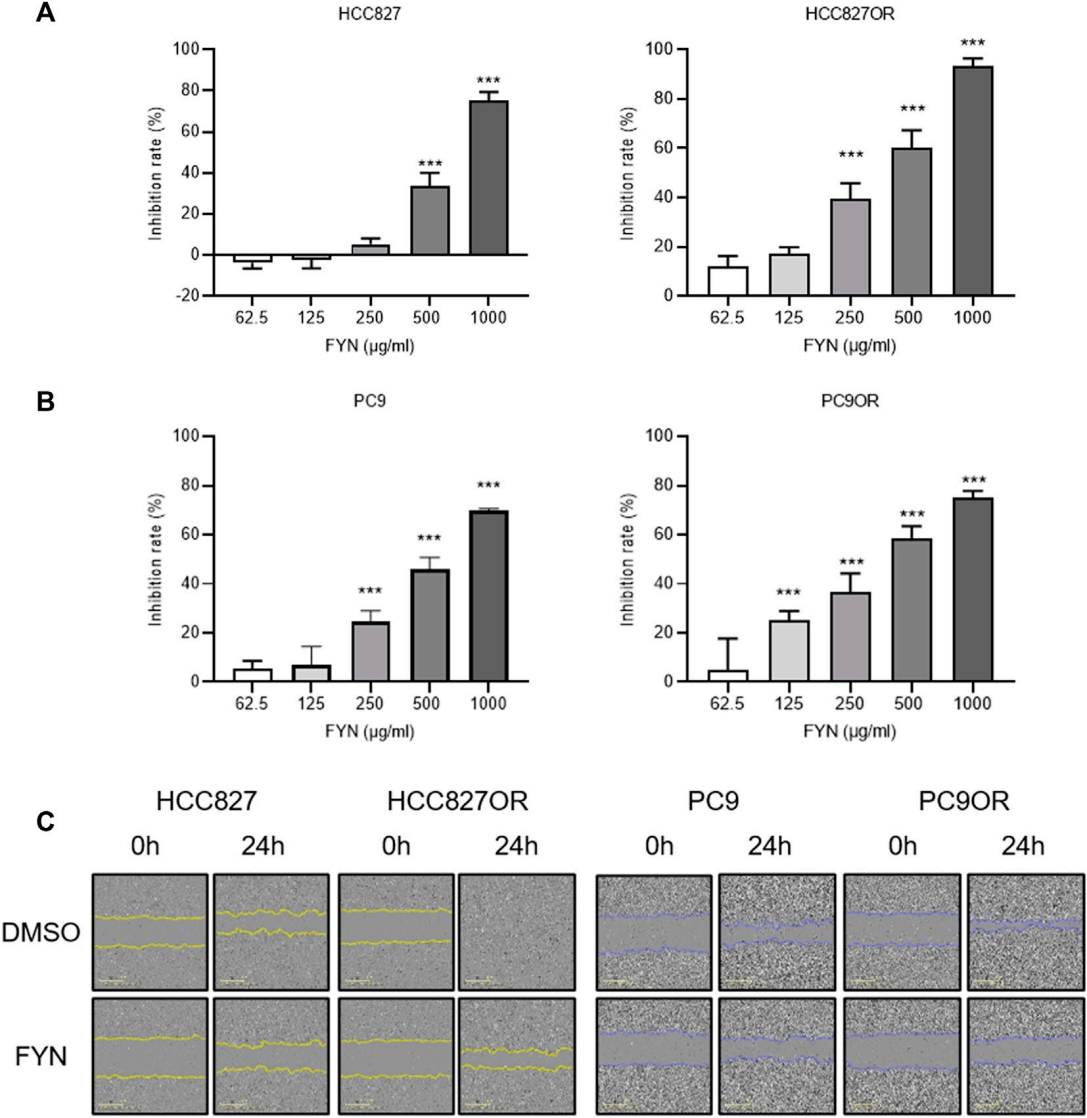
Effects of FYN on lung cancer cell proliferation and migration. **(A,B)** CCK8 results. HCC827 or HCC827OR cells **(A)** and PC9 or PC9OR cells **(B)** were treated with different concentrations of FYN for 48 h. **(C)** Cell migration was detected *via* wound healing assays. HCC827 or HCC827OR cells were treated for 24 h with 250 μg/ml FYN, and PC9 or PC9OR cells were treated for 24 h with 125 μg/ml FYN. *p < 0.05, **p < 0.01, ***p < 0.001.

The rates of PC9OR and HCC827OR cell inhibition were significantly reduced compared to parental cells after 48 h of osimertinib intervention, in a dose-dependent manner (Supplementary Figure S1A). Protein levels of PCNA in HCC827OR and PC9OR cell lines were higher than in the parental counterpart (Supplementary Figure S1B), indicating enhanced cellular proliferation and invasion. FYN preferentially suppressed cellular proliferation and migration in osimertinib-resistant HCC827OR and PC9OR cells, and it had higher potency and efficacy than in the parental HCC827 and PC9 cells (Figures 1A–C).

### Synergistic effect of FYN with osimertinib on resistant lung cancer cell lines

**FIGURE 2:**
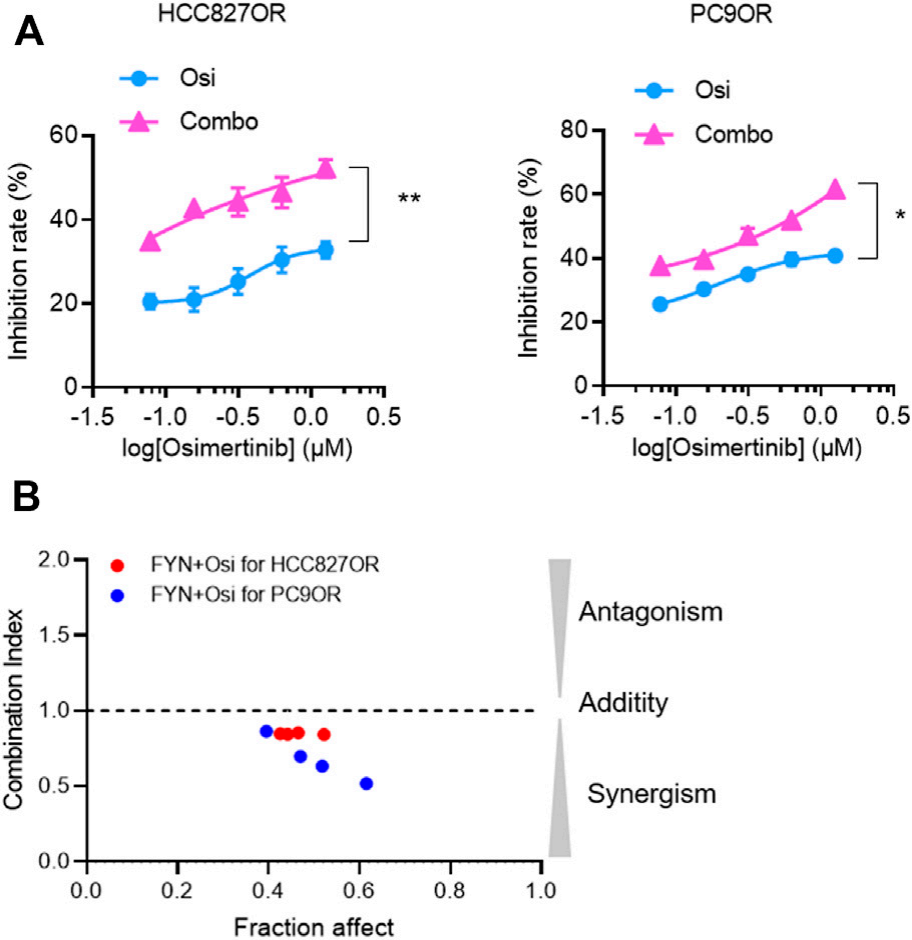
Synergistic effects of FYN with osimertinib on osimertinib-resistant NSCLC cells lines. **(A)** HCC827OR cells were treated for 48 h with 4 μM osimertinib alone, or in combination with 250 μg/ml FYN. PC9OR cells were treated for 48 h with 6 μM osimertinib alone, or in combination with 500 μg/ml FYN. **(B)** Synergistic effects of 250 μg/ml FYN on HCC827OR cells (red circles), or 500 μg/ml FYN on PC9OR cells (blue circles), combined with various dose of osimertinib. CI (combination index) value was calculated as described in Materials and Methods. Osi, osimertinib; FYN, Feiyanning Formula; Combo, combination of Feiyanning Formula and osimertinib. *p < 0.05, **p < 0.01.

**FIGURE 3:**
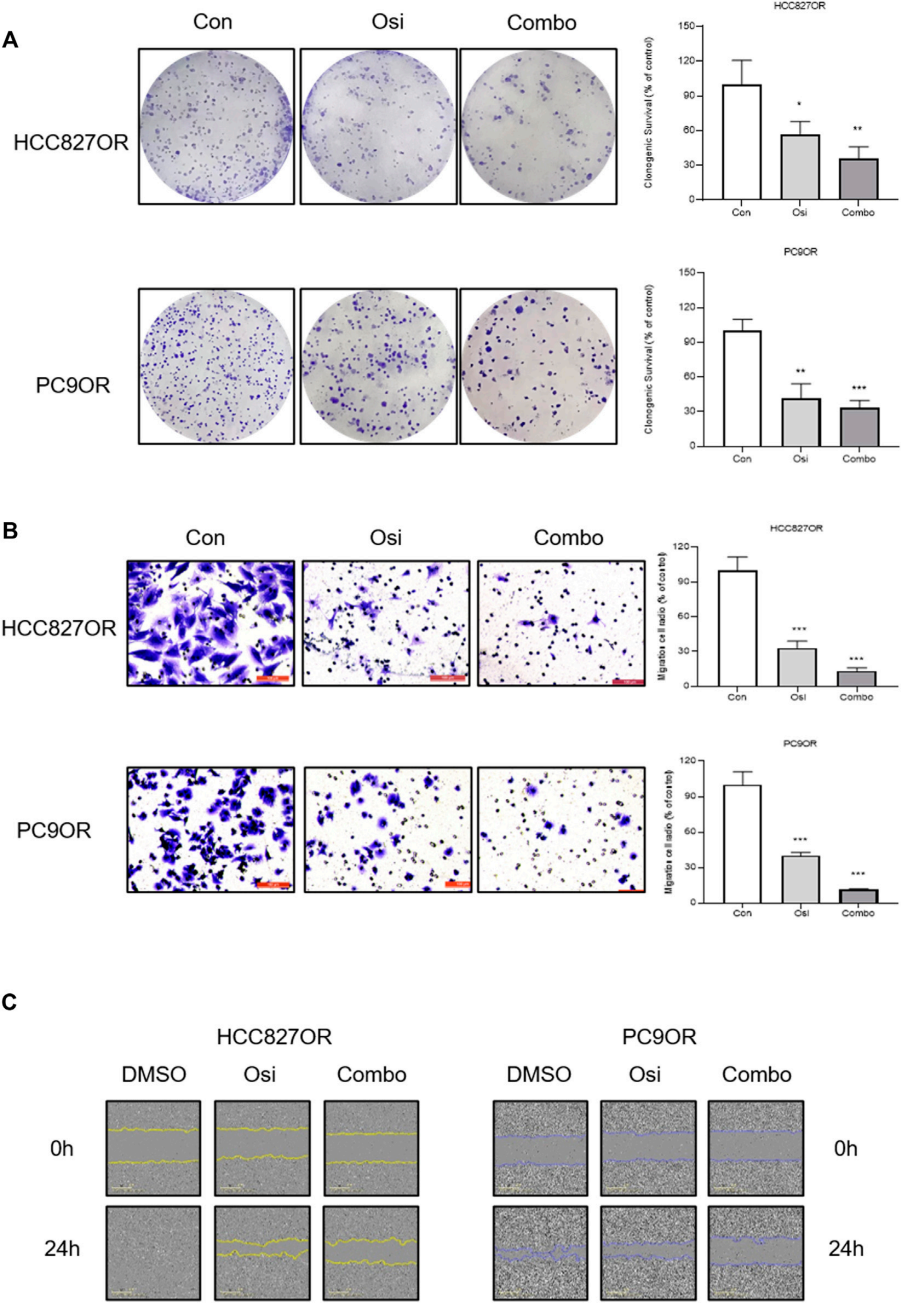
The synergistic effect of FYN with osimertinib on cell proliferation and migration. Resistant HCC827 cells were treated with 1 μM osimertinib alone or in combination with 250 μg/ml FYN. Resistant PC9 cells were treated with 1 μM osimertinib alone or in combination with 125 μg/ml FYN. **(A)** Clonogenic assays results of HCC827OR and PC9OR and the cells were treated for 14 days. **(B)** Cell migration was detected by transwell assays or **(C)** wound healing assays for 24 h. Con, control; Osi, osimertinib; Combo, combination of Feiyanning Formula with osimertinib. *p < 0.05, **p < 0.01, ***p < 0.001.

To investigate the synergistic effect of FYN with EGFR-TKI, CCK-8 assays and colony formation assays were performed. The effects of the combination of FYN and osimertinib on the viability of HCC827OR and PC9OR cells were assessed. The combination exhibited more significant inhibition compared with osimertinib alone (Figure 2A, Supplementary Figure S1C). The CI was less than 1.0 (Figure 2B), suggesting a synergistic effect of FYN and osimertinib in resistant cell lines. Colony formation assays indicated that the combination substantially inhibited the long-term proliferation of HCC827OR and PC9OR cells (Figure 3A). Thus, the combination exhibited significant inhibition of the viability of drug-resistant cells.

Transwell assays and wound-healing assays were performed to investigate the effects of the combination of FYN and osimertinib on migration and invasion. Cell migration was enhanced in HCC827OR and PC9OR cell lines compared to the parental lines (Figure 1C). Cell migration was inhibited after 24 h of treatment with the combination of FYN and osimertinib in HCC827OR and PC9OR cell lines, compared to control and osimertinib alone (*p* < 0.05) (Figure 3B). Consistent results were obtained in wound-healing assays (Figure 3C).

### FYN downregulated mRNA expression in the Wnt/β-catenin pathway

**FIGURE 4:**
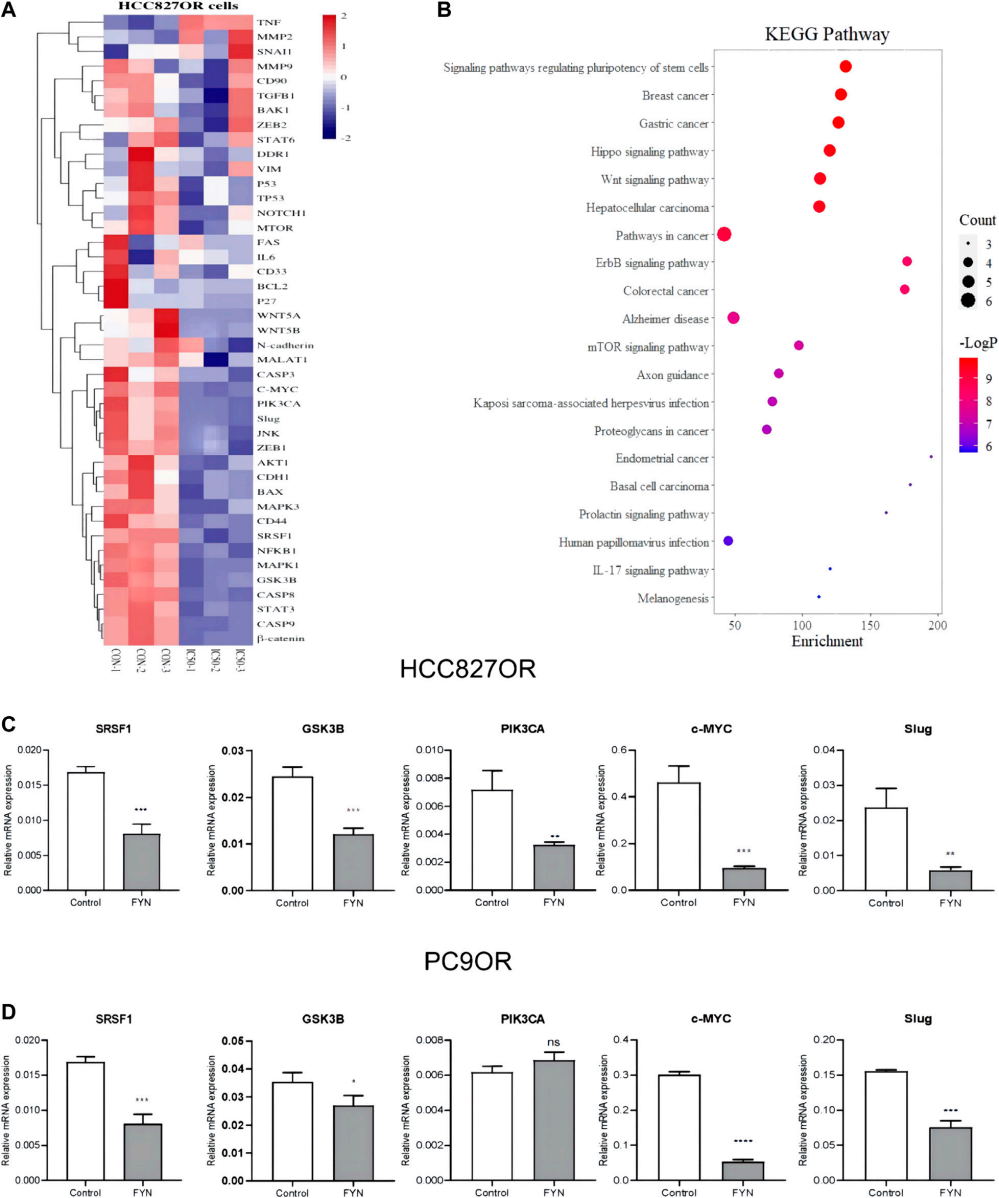
FYN downregulated the mRNA levels of genes enriched in the Wnt/β-catenin pathway. HCC827OR cells treated with FYN at IC_50_ for 48 h were harvested for analysis **(A)** Heatmap of target mRNA expression in control HCC827OR cells and FYN-treated HCC827OR cells. **(B)** KEGG analysis mostly enriched differentially expressed genes in the Wnt/β-catenin pathway. **(C and D)** mRNA levels in HCC827OR cells **(C)** and PC9OR cells **(D)** were measured by qPCR. FYN, Feiyanning Formula. *p < 0.05, **p < 0.01, ***p < 0.001.

RT-qPCR array analysis was performed after 48 h of treatment with an IC_50_ dose of FYN in HCC827OR cell lines. mRNA levels of c-MYC, GSK3B, PIK3CA, Slug, and SRSF1 were downregulated (Figures 4A,C). c-MYC, GSK3B, Slug, and SRSF1 were downregulated after 48 h of exposure to FYN in PC9OR cell lines (Figure 4D). KEGG analysis of the genes above indicated they were primarily enriched differentially expressed genes in the Wnt/β-catenin pathway (Figure 4B).

### FYN reduced the expression of SRSF1, GSK3B, and β-catenin

**FIGURE 5:**
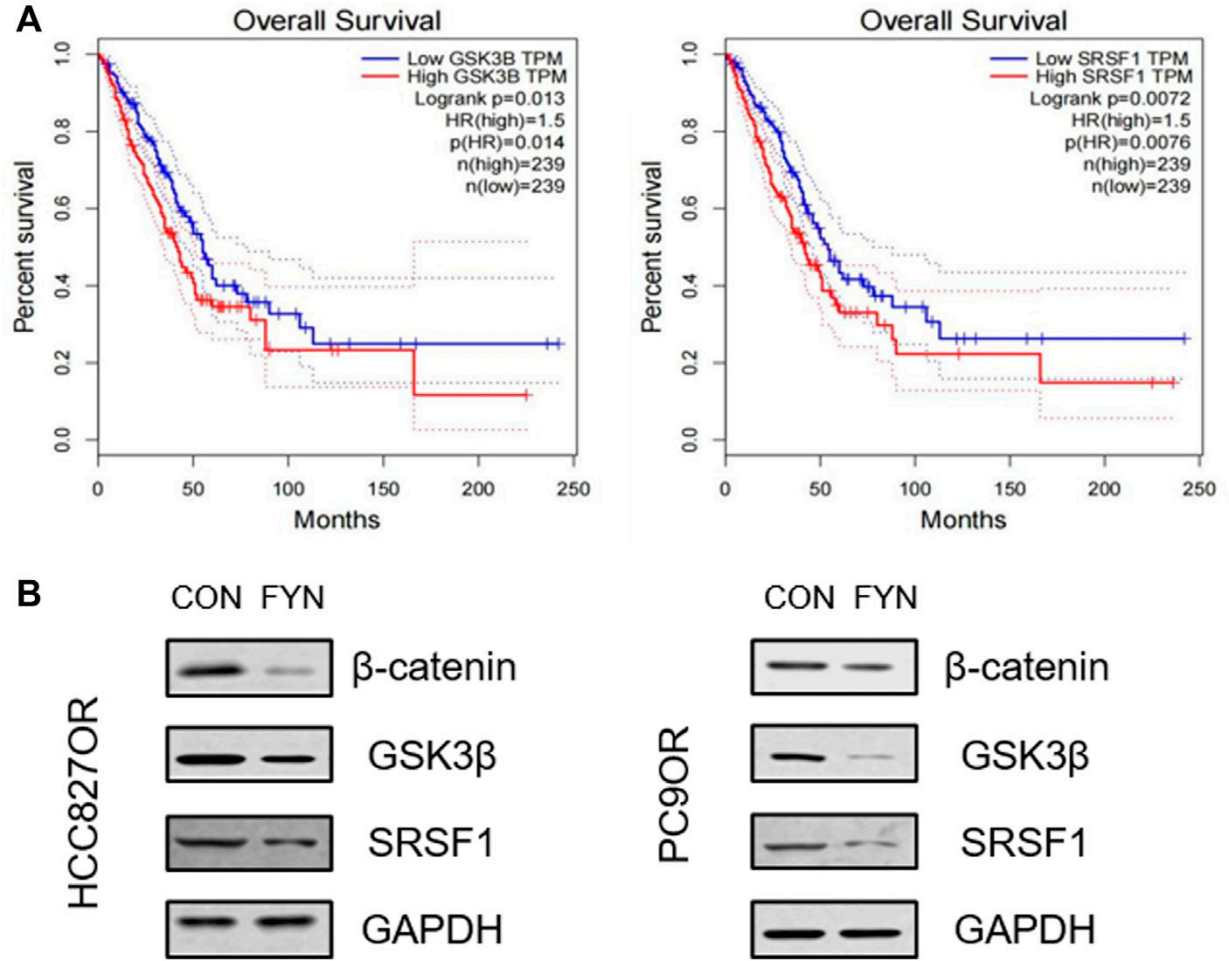
FYN reduced SRSF1, GSK3β, and β-catenin protein levels. **(A)** Correlations between overall survival in LUAD patients and GSK3β and SRSF1 expression, analyzed using the GEPIA website. **(B)** HCC827OR cells were treated with FYN at IC_50_ or isovolumetric 1640 medium for 48 h for analysis. Protein levels of SRSF1, GSK3B, and β-catenin were determined by western blotting. Con, control; FYN, Feiyanning Formula.

The differentially expressed genes were screened *via* the GEPIA website (http://gepia.cancer-pku.cn/). SRSF1 and GSK3B were filtered through “overall survival”, and high expression was associated with a poorer prognosis than low expression in a subset of lung adenocarcinoma patients (Figure 5A). Western blotting results indicated that FYN could significantly downregulate SRSF1 and GSK3B protein expression in HCC827OR and PC9OR cells. Since the Wnt/β-catenin pathway mainly regulates downstream signaling pathways with β-catenin as the core node, we investigated the expression of β-catenin in HCC827OR and PC9OR cells after FYN intervention. FYN could downregulate β-catenin, suggesting that the Wnt/β-catenin pathway may be involved in FYN’s effects on osimertinib resistance (Figure 5B).

### FYN suppressed tumor growth *in vivo*

**FIGURE 6:**
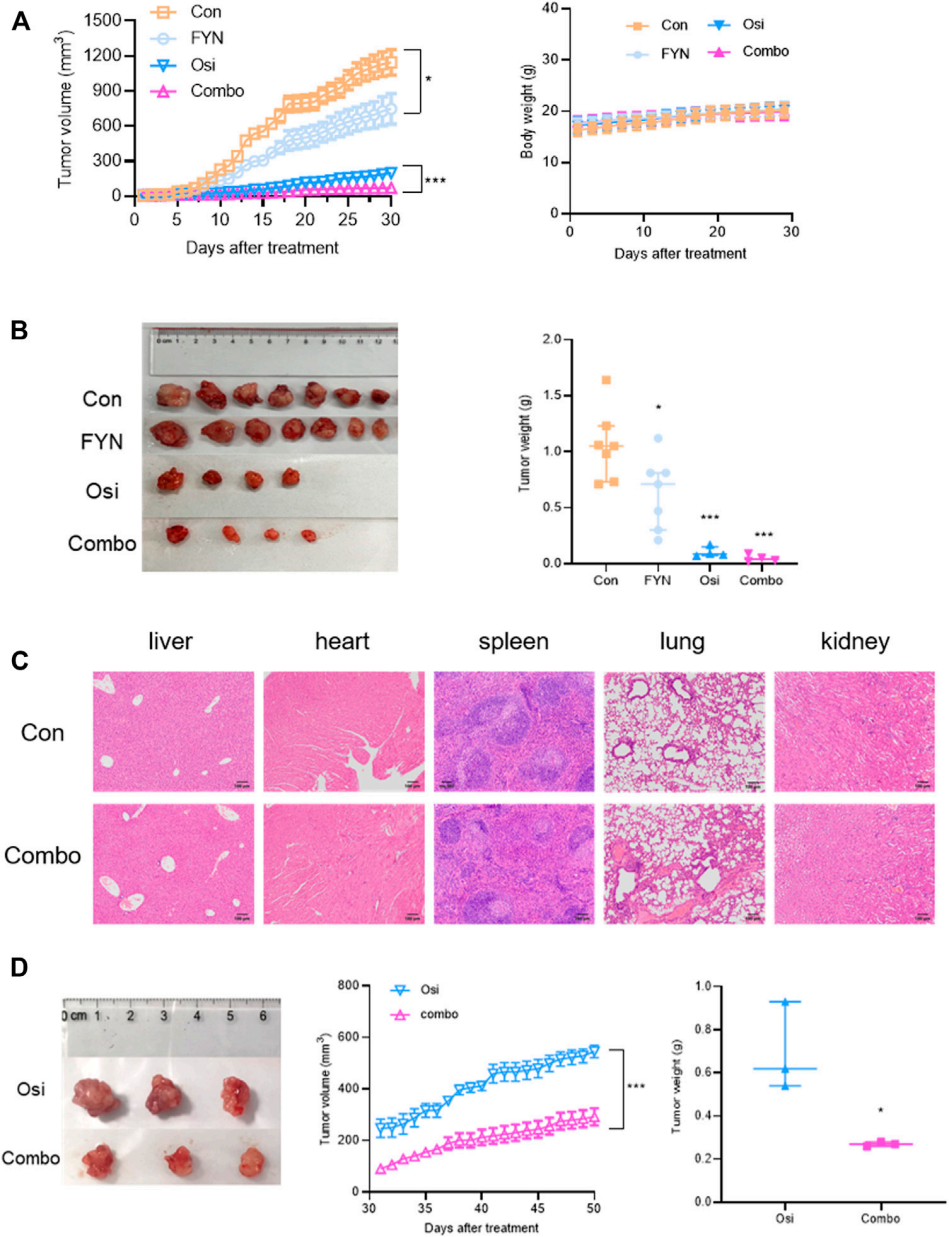
FYN combined with osimertinib suppressed tumor growth in mouse xenograft models of lung adenocarcinoma. **(A)** Tumor growth curve and body weight of subcutaneous HCC827OR xenografts (*n* = 7). **(B)** Mice in the control group (*n* = 7), FYN group (*n* = 7), osimertinib group (*n* = 4) and combination group (*n* = 4) were anaesthetized to determine tumor volumes and weights 30 days after treatment. **(C)** Organs of mice in the control group and the combination group were harvested 30 days after treatment and stained with hematoxylin and eosin. **(D)** Tumor growth curves and tumor weights of 3 mice in the osimertinib group and 3 mice in the combination group. Con, control; FYN, Feiyanning Formula. Osi, osimertinib; Combo, combination of Feiyanning Formula and osimertinib. *p < 0.05, **p < 0.01, ***p < 0.001.

To validate the synergistic effects of FYN with osimertinib *in vivo*, murine subcutaneous xenograft experiments were performed. In HCC827OR xenografts (Figures 6A,B, Supplementary Table S2), compared with the control group tumor growth was inhibited in the other three groups, particularly in the combination group (*p* < 0.05). No lesions were observed in heart, kidney, liver, spleen, or lung tissues in the combination group (Figure 6C). Considering that the tumor volume of the control group met the ethical requirements, 7 mice in the control group, 7 mice in the FYN group, 4 mice in the osimertinib group, and 4 mice in the combination group were terminated after 30 days of treatment. Due to the small volumes of tumors, 3 mice in the osimertinib group and 3 mice in the combination group were retained for further observation under prolonged intervention. Tumor volume increased significantly after 20 days in the osimertinib group, which may indicate the development of resistance. Compared with the osimertinib group, tumor growth was significantly inhibited in the combination group, suggesting that FYN could partly against the adapt resistance of osimertinib (Figure 6D).

## Discussion

Most drug resistance development occurred after treatment with former generations of EGFR-TKIs, with a progression-free survival of 10–14 months (Zhang et al., 2019; Wu et al., 2020). T790M mutation was believed to be the culprit. Osimertinib has been used as a first-line treatment in NSCLC patients with EGFR Mutations due to significant therapeutic effects on both common EGFR mutations (Del19 and L858R) and T790M resistance mutations (Popat, 2018). However, the development of resistance remains inevitable (Du et al., 2021). Resistance mechanisms such as C797S site mutations, MET and HER2 amplification, bypass activation, and conversion to NSCLC are reportedly involved (He et al., 2021). Traditional Chinese Medicine combined with targeted therapy can reportedly prolong survival and delay drug resistance (Zhang et al., 2018; Jiao et al., 2019; Lu et al., 2021). Abundant herbal monomers combined with osimertinib had the effect of delaying drug resistance. The mechanisms involved included tumor stem cell inhibition, regulation of reactive oxygen species, and alteration of downstream pathways (Hu et al., 2020; Lai et al., 2021). In contrast, research on herbal formulas remains at blurred stages. Therefore, it is necessary to determine the mechanisms of FYN in drug resistance regulation.

FYN contains 11 herbs and exhibits anti-cancer effects (Xu et al., 2011). Although FYN can inhibit the protective autophagy induced by cisplatin in NSCLC cells (Zheng et al., 2021), whether it can modulate osimertinib resistance in NSCLC remains unclear. In the current study, FYN inhibited the viability of HCC827OR and PC9OR cells in a dose-dependent manner. FYN combined with osimertinib could enhance the inhibition of cell viability, inhibit colony formation, and suppress invasion and migration. These observations indicated that FYN had synergistic effects.

Numerous studies have shown that the Wnt/β-catenin pathway is a major contributor to the development of drug resistance to EGFR-TKIs (Wang et al., 2020; Yan et al., 2022). The Wnt signaling pathway is involved in a variety of physiological processes including cell proliferation, developmental metabolism, and cell migration. Moreover its abnormal activation is associated with tumor growth (Koni et al., 2020; Yu et al., 2021; Liu et al., 2022). In the current study, the differential genes were screened using PCR array. According to KEGG analysis, these genes are primarily enriched in the Wnt/β-catenin pathway. FYN downregulated the expression of SRSF1 and GSK3B. SRSF1 is a multifunctional protein involved in RNA metabolism. It induces a cancer cell cycle, proliferation, apoptosis, tumorigenic angiogenesis, and metastasis (Sokół et al., 2017). Upregulation of SRSF1 activates the Wnt pathway, promoting tumor growth (Malakar et al., 2017; Zhang et al., 2021), which may be related to the accumulation of β-catenin proteins (Fu et al., 2013). Glycogen synthase kinase-3β is a serine/threonine kinase that participates in various signaling pathways including Wnt/β-catenin. It also has anti-tumor effects (Domoto et al., 2016; Lin et al., 2020). This is consistent with results in the current study, implying that the Wnt/β-catenin pathway is the core pathway involved in FYN against osimertinib resistance.

## Conclusion

The combination of FYN and osimertinib may inhibit the cellular activities of osimertinib-resistant cells *in vitro* and *in vivo*, leading to anti-tumor effects. In this study, FYN downregulated the mRNA expression of SRSF1, β-catenin, and GSK3B. FYN delayed osimertinib resistance by regulating the Wnt/β-catenin pathway. The current study provided scientific evidence for the application of Chinese herbal medicine on NSCLC treatment, particularly in combination with EGFR-TKI.

## Data Availability

The raw data supporting the conclusion of this article will be made available by the authors, without undue reservation.

## Supplementary material

SUPPLEMENTARY FIGURE S1
Effects of osimertinib on the proliferation of different lung cancer cell lines. **(A)** Effects of osimertinib on HCC827 and PC9 osimertinib sensitivity in parental (white) and osimertinib-resistant (gray) cells. Cells were treated with various concentrations of osimertinib for 48 h and tested *via* the CCK8 assay. **(B)** Protein levels of PCNA were measured *via* western blotting. **(C)** CCK8 results. HCC827OR cells were treated with 4 μM Osimertinib with FYN and PC9OR cells were treated for 48 h with 6 μM osimertinib with FYN for 48 h. ∼P, parental cell lines; ∼OR, osimertinib-resistant cell lines. **p* < 0.05, ***p* < 0.01, ****p* < 0.001.
