# Supplementary figures and images for: Feiyanning formula modulates the molecular mechanism of osimertinib resistance in lung cancer by regulating the Wnt/β-catenin pathway
Source: Front Pharmacol. 2022 Nov 29;13:1019451. doi: 10.3389/fphar.2022.1019451 (PMC9745155; doi:10.3389/fphar.2022.1019451)

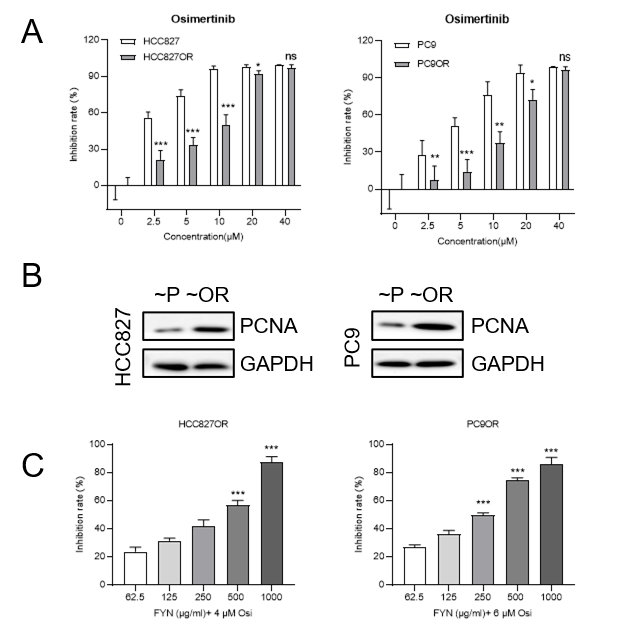

Supplement: Supplementary file 2 [file Image1.TIF]
